# The Impact of Climate on the Spread of Rice to North-Eastern China: A New Look at the Data from Shandong Province

**DOI:** 10.1371/journal.pone.0130430

**Published:** 2015-06-30

**Authors:** Jade d’Alpoim Guedes, Guiyun Jin, R. Kyle Bocinsky

**Affiliations:** 1 Department of Anthropology, Washington State University, Pullman, Washington, USA; 2 School of History and Culture, Shandong University, Jinan, Shandong, China; Chinese Academy of Sciences, CHINA

## Abstract

Moving crops outside of their original centers of domestication was sometimes a challenging process. Because of its substantial heat requirements, moving rice agriculture outside of its homelands of domestication was not an easy process for farmers in the past. Using crop niche models, we examine the constraints faced by ancient farmers and foragers as they moved rice to its most northerly extent in Ancient China: Shandong province. Contrary to previous arguments, we find that during the climatic optimum rice could have been grown in the region. Climatic cooling following this date had a clear impact on the distribution of rice, one that may have placed adaptive pressure on rice to develop a temperate phenotype. Following the development of this temperate phenotype, rice agriculture could once again become implanted in select areas of north-eastern China.

## Introduction

Despite its high productivity, rice was a challenging crop to move into environments outside of its original center of domestication. Rice requires not only special accommodations in terms of water management systems, but also high temperatures necessary to sustain its growth. What factors facilitated or inhibited the spread of rice agriculture outside of its original centers of domestication are not well understood. Climate—and specifically long-term temperature changes—could have played an important role in the spread of this crop throughout eastern Asia. Here, we model the spatial and temporal crop niche of two varieties of rice in the Shandong Province of eastern China over the last 8,000 years. Similar crop niche models have recently been developed for the American Southwest [1], for Europe [2] and for Southwest China [3] and the Tibetan Plateau [4]. The application of systematic archaeobotany to Shandong Province provides crucial evidence for documenting how this spread took place in a region located at the very limits of the growing niche of rice. Using a crop niche modeling, we demonstrate that Shandong province provided sufficient temperatures for the growth of tropical *O. japonica* rices only during the warmer temperatures of the Holocene climatic optimum. As temperatures grew cooler, the territory in which these rices could be grown underwent a substantial decline. We argue that areas of southern Shandong, which lie on the very border of tropical *O. japonica* viability, may have placed adaptive pressure on rices that lead to the development of temperate varieties of *O. japonica*.

## The Archaeobotanical Evidence from Shandong

**Fig 1:**
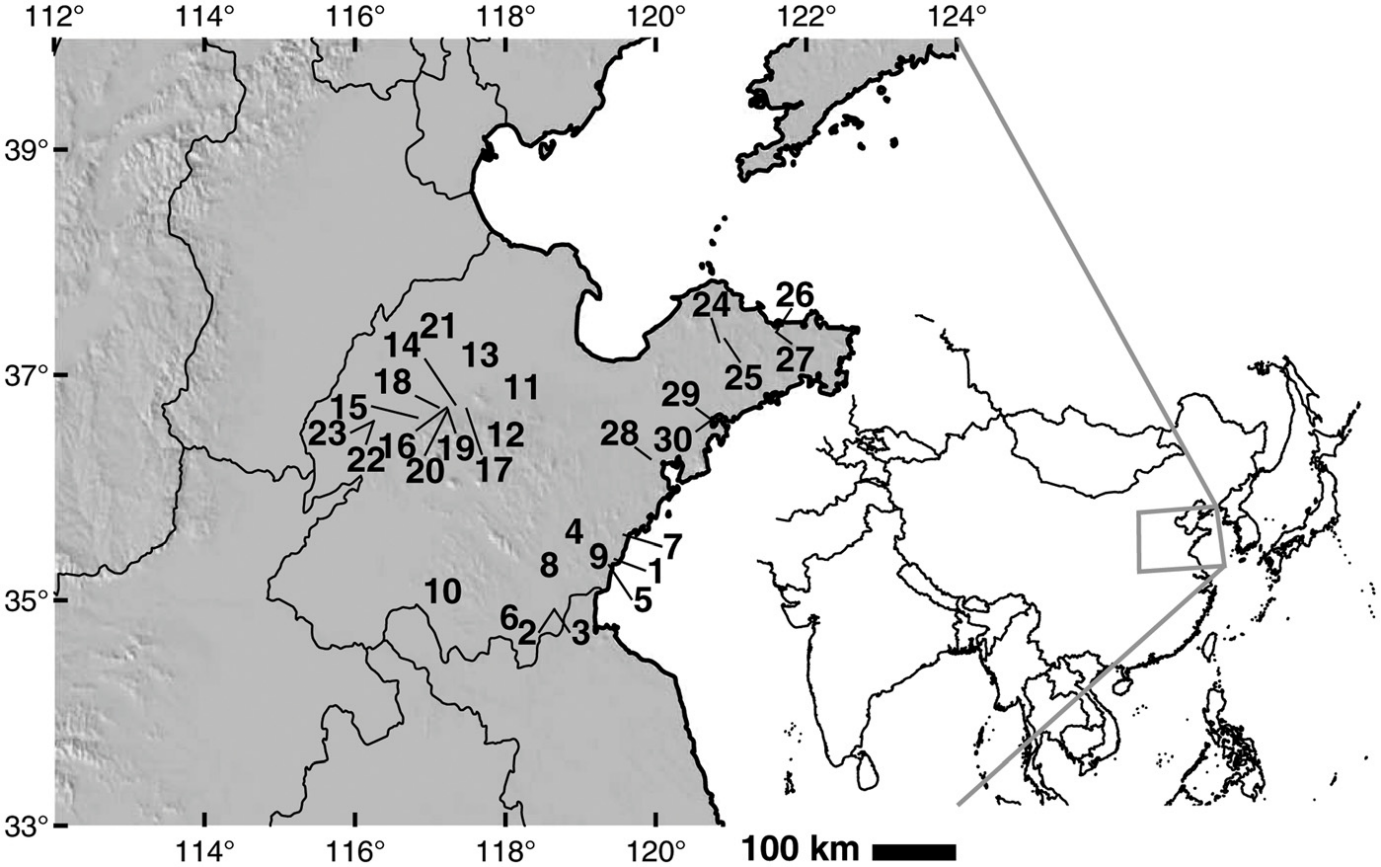
Sites discussed in the text. 1: Rizhao Nantungling; 2: Linshu Dongpan (Yangshao); 3: Linshu Dongpan (Longshan); 4: Rizhao Xujiacun; 5: Rizhao Yaowangcheng; 6: Cangshan Houyuanguanzhuan; 7: Liangchenzhang; 8: Juxian and Jiaozhou; 9: Zhucheng Xuejiazhuang; 10: Zhuanglixi; 11: Tongling; 12: Zhangdian Fangjia; 13: Gaoqing Chenzhuang; 14: Xihe; 15: Yuezhuang; 16: Jinan Pengjia; 17: Ma’an; 18: Daxinzhuang; 19: Jinan Cuimazhuang; 20: Jinan Tangye; 21: Laoling Yinjie; 22: Chiping Jiaochangpu and Taizigao sites; 23: Chiping Longshan sites; 24: Yangjiaquan; 25: Yuanjiaquan; 26: Yantai Miaohou; 27: Yantai Zhaogezhuang; 28: Jiaozhou Zhaojiazhuang; 29: Beiqian (Dawenkou); 30: Beiqian (Chunqiu). Map relief from ETOPO1 [6]; country and province outlines from Natural Earth (naturalearthdata.com).

Shandong province is located in North Eastern China (Fig 1). The interior central portion of the province is composed of highlands that rise to roughly 300 masl. Surrounding these highlands are low-lying plains. The most eastward part of the province is a peninsula that extends into the ocean. Elevations on the peninsula are higher in the north and lower in the south. Both precipitation and temperature conditions vary across the province. The southeastern part of the province receives the most rain (900 mm per year), while the northwest part of the province receives the least (550 mm per year). Northern Shandong peninsula is on average cooler (2400 GDD per year on a 10°C base) than the interior (2500–2800 GDD on a 10°C base) and southern part of the province (roughly 3000 GDD on a 10°C base). The main crops grown in this area today are wheat, corn, peanut and soybean. Only small quantities of rice are grown in this area today, and only in the areas of southern Rizhao, Jining and Jinan [5].

Shandong province is situated at quite some distance from the original center of domestication of rice; however, because of its proximity to Korea and Japan, data from this province is crucial to understanding how rice agriculture eventually moved into other areas of East Asia. With the exception of a single find of rice phytoliths in Liaoning province [7], Shandong appears to have constituted the most northern frontier for the distribution of rice in China, making it an important area to study the challenges faced by rice agriculturalists as they moved north. Genetic evidence has confirmed that *O. japonica* rices were domesticated in China, likely in one or two centers of domestication in the middle and/or lower Yangtze river valley [8]. Early rice dating to 6000–5000 cal. BC has been discovered in central Shandong at sites of the Houli Culture [9, 10]. Finds from the Yuezhuang site, located near the central Shandong highlands were directly dated to 6000–5000 cal. BC [9]. Rice grains were present alongside broomcorn and foxtail millet. No spikelet bases or awn remains were found alongside these carbonized rice grains and as a result, it is unclear if these grains of rice were cultivated or wild [9]. More recently, new evidence for early rice in Shandong has been excavated from the site of Xihe located near Yuezhuang in the Shandong highlands [10]. Rice grains together with phytoliths from rice husks overwhelm the assemblage at the site although very small numbers of foxtail millet were present. It is unclear if these grains were wild or domesticated as no spikelet bases were unearthed from this site. Phytoliths have been unearthed at another site, Yuhuangding in Jining that dates to 5000 cal. BC, however no grains have been unearthed [11]. Animal remains at Houli sites show that the inhabitants of these sites appear to have been reliant on hunting and fishing [10]. Aside from potential domestic pig (although this may also be wild), the majority of animal remains at Houli sites are composed of deer, fish, birds and various types of clams and shells [10]. Isotopic studies have been carried out at Houli period sites and indicate a mixed C3/C4 diet that included at least some C4 plants such as millet but also some C3 plants [12]. This is not altogether unsurprising as the majority of wild plants found in the area are C3. These finds are far from the original centers of domestication of *O. japonica* and are situated much further north than expected. It is thus unclear if this rice was locally grown or was imported to the site from another from a location further to the south [9]. This same concern has been raised for finds of rice in the Huai River Valley [13].

Between 5000–2600 cal. BC, only few finds of rice have been uncovered and these have all been retrieved from flotation samples of small size and less than secure provenience. Most finds are confined to southern Shandong. Two grains of rice (out of a total of four recovered grains: one broomcorn and one foxtail millet) were unearthed at the Linshu Dongpan site in Southern Shandong that dates to 4030–3820 cal. BC [14]. Where systematic flotation has been carried out such as at Beiqian (early to middle Dawenkou Culture: 3600–2900 cal. BC), results show a subsistence system based entirely on foxtail and broomcorn millet, and no rice grains were recovered [15, 16]. At Xujiacun (3000–2500 cal. BC) in southern Shandong, only six seeds were recovered from flotation samples, only one of which was rice. No direct dating was carried out on these seeds. A survey in the Juxian and Jiaozhou (3000–2400 cal. BC) area of the Haidai region provided a total only of 9 seeds were recovered, only two of which were rice. A large percent of rice phytoliths were unearthed from these samples, however given that these came from a survey it is possible that these could have been modern [17]. Again, none of these finds have been directly dated.

Rice again forms a larger part of the archaeobotanical assemblage in Shandong during the Longshan period (c. 2600 BC). At the site of Liangchengzhen, rice overwhelms the assemblage [18]. Tiny quantities of wheat were also unearthed at the site, along with broomcorn millet, foxtail millet, soybean and adzuki bean [18]. In far southern Shandong, rice re-appears in Longshan layers of the Linshu Dongpan site, although now in higher proportions and numbers. Rice remains also constitute over 50% of the assemblage at the Zhaojiazhuang site. In addition to containing remains of rice caryopses, physical evidence for rice cultivation at this site is also present in the form of paddy remains that have been unearthed at the site [19]. The presence of rice as a primary domesticate appears to last roughly 600 years. Finds of rice continue in the region, but decline in number following 2000 cal. BC [20]. This decline in numbers of rice grains found on sites also coincides with a decrease in the number of sites overall. Many of the late Longshan sites appear to be abandoned at around 2000 cal. BC. The reason for this decline are unclear although climatic factors have been implicated in this reorganization of settlement patterns in other areas of China [21, 22].

**Fig 2:**
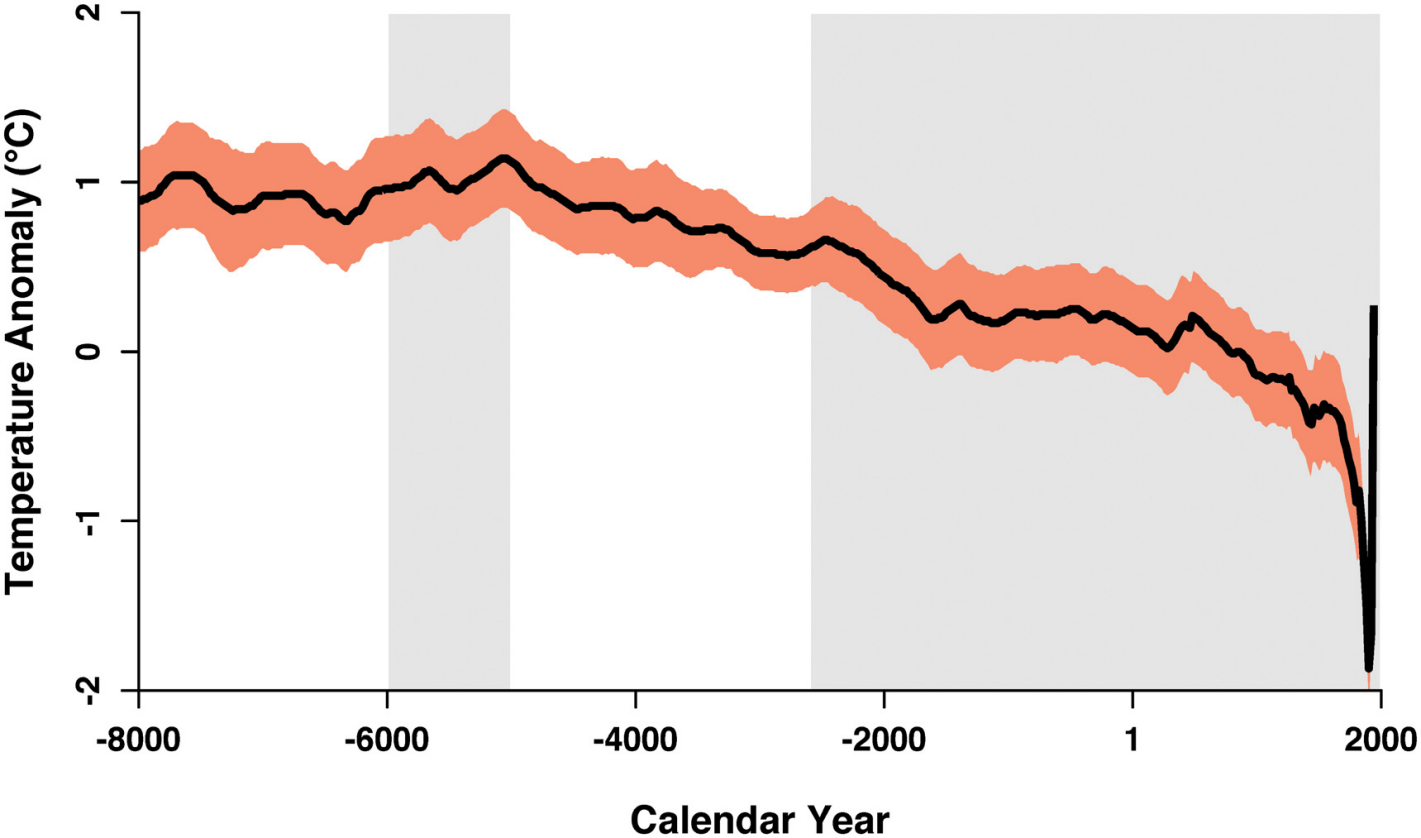
The northern hemisphere temperature anomaly and presence of rice in Shandong during the Holocene. Temperature anomaly (black line) and confidence intervals (orange band) are as reported by Marcott [23]. Gray regions indicate periods of probable rice cultivation in Shandong.

In summary, during the earlier part of the Holocene, reliable finds of rice in Shandong are present in sites of the Houli culture that date to between roughly 6000–5000 cal. BC (Fig 2). Because of their distance from rices original center of domestication, the nature of these remains have been disputed. While initially suspected to be later intrusions, radiocarbon dates have confirmed that these finds are indeed early Holocene in date [9]. Only a few sparse finds of rice have been uncovered following this date. As none of these finds have been directly dated and as many were recovered from surveys, it is unclear if these are reliable evidence for the exploitation of rice. Rather it seems that between c. 5000–2600 cal. BC, rice is absent from the Shandong peninsula. Rice then re-appears on the peninsula during the Longshan period (c. 2600 cal. BC). Despite evidence derived from growing numbers of sites, it is still unclear why rice is present so far north of its original center of domestication between 6000–5000 cal. BC. The reasons for the long abandonment of rice, followed by its reintroduction are equally unclear, although climatic reasons have been implicated. Our understanding of the patterns underlying the spread of rice agriculture across China has been hampered by lack of models capable of predicting the niche of rice in the past. We employ such a model with the aim of answering two questions: Was early Holocene rice in Shandong locally grown or rather traded into this site? What were the factors that finally allowed rice agriculture to become implanted once again on the peninsula during the Longshan period?

## Materials and Methods

An understanding of plant phenology (the relationship between biological events in an organisms life cycle and the climate in which they live) allows us determine plants’ physiological limitations [24]. There are a number of key abiotic factors involved in plant growth and development: nutrients, soil, air (CO_2_), sunlight for photosynthesis, and sufficient temperatures for enabling growth. Adequate temperature is essential to rice growth. Varieties of rice domesticated in China were originally tropical crops and require in the past as they do today, large amounts of heat in order to complete its growth cycle and many varieties require temperatures in excess of 25°C in order to initiate germination. Temperature thus forms an important limiting factor for rice growth.

We use growing degree days (GDDs; a measure of accumulated heat) to define limits of the thermal niche of rice [25, 26]. We do not model precipitation, as East Asian rices were often grown in lowland or paddy environments and were therefor more subject to niche construction and not directly dependent on rainfall. However, it would be important to model the precipitation niche for crops not grown in wetlands or those grown in more arid environments.

Calculations of growing degree days (GDD) assume that there is a base temperature (*T*_base_), below which an organism grows very slowly or not at all; crop growth increases with temperatures above the baseline. Different crops (and often different varieties of the same crop) have different baseline temperatures and requirements of accumulated GDD in order to achieve maturity. For instance, while wheat, barley and rye have baseline temperatures of 0°C, most varieties of rice have baseline temperatures of 10°C. GDD can be calculated for each individual day using the following formula:

$$\text{GDD}=\frac{T_{\text{max}}+T_{\text{min}}}{2}-T_{\text{base}}$$ (1)

where *T*_min_ and *T*_max_ are the minimum and maximum daily temperature, respectively. We apply a now-standard correction that up-corrects *T*_min_ and *T*_max_ to *T*_base_, such that

$$\begin{array}{c} \text{if}\;\;T_{\text{max}}<T_{\text{base}},\;T_{\text{max}}=T_{\text{base}}\\ \text{if}\;\;T_{\text{min}}<T_{\text{base}},\;T_{\text{min}}=T_{\text{base}} \end{array}$$

These GDD values are then summed over the course of an entire calendar year to obtain a yearly GDD total.

Genetic studies have shown that rice clusters into two separate complexes: *O. indica* and *O. japonica* [27, 28]. It is now understood that *O. indica* and *O. japonica* rices were domesticated separately: *O. japonica* from *O. rufipogon* in China and *O. indica* from *O. nivara* in the Indian subcontinent. Within *O. japonica*, temperate japonica species, tropical *O. japonica* species and aromatic sub populations are grouped together. Most shared genetic material occurs between temperate and tropical *O. japonica*[29, 30], suggesting that these groups are selections from a single genetic pool that have been adapted to different ecological conditions [31]. In a 2005 study, the alleles at 15 monomorphic loci in temperate *O. japonica* were identical in size to tropical *O. japonica*, leading one to believe that temperate japonica rices may have been derived from tropical *O. japonica* [27]. This hypothesis is also supported by evidence that the average standardized allele size was greater in temperate japonica than in tropical *O. japonica*. One explanation for this is that adaptive pressure put on temperate varieties may have encouraged higher numbers of mutations [27, 31]. It is likely that early domesticates were tropical varieties of *O. japonica* [32].

For several reasons, the modern distribution of rice cannot be used to infer the areas that rice was able to occupy in the past. Rices grown in China have undergone a series of improvements in breeding that have adapted varieties to cooler environments during the historic period. In addition to the introduction of *O. indica* c. 100 AD, other varieties of rice have been introduced to China over the course of history. Varieties of early ripening rices were introduced to China during the Song dynasty. Champa (or zhan-cheng) an early maturing indica rice was introduced from Vietnam c. AD 1012 [33–35]. This variety could be planted in the highlands and is reported as having a growing of 100–110 days [36]. The introduction of Champa rice was a huge success and soon was grown in over 90% of the fields in the lower Yangtze [37]. Introduction of Champa rice lead to a huge increase in rice productivity in China allowing rice to move into upland areas and for double cropping and even triple cropping to be carried out in some regions. Historical sources around the time of the Song dynasty indicate that prior to the introduction of Champa-type short ripening varieties, traditional varieties of rice required over 150–165 days for ripening of early varieties, and between 180–200 days for late varieties [36]. This may mean that growing degree day requirements were even higher than we were able to estimate for these ancient varieties. During the Ming and Qing dynasties (AD 1300–1900), agricultural manuals suggest that elite mutants were selected for traits such as large grains and short season growth [38]. In addition to historical gains in rice production, rice grown in China has undergone substantial changes in productivity and growing season since the 1950’s. During the 50’s dwarf varieties of rice allowed growth in a wide range of ecological conditions were developed from crossing with varieties from Japan, Korea and Italy [35]. Zajiao or hybrid rice was developed by Yuan Longping during the 1970’s and now is grown in well over 50% of fields in China [35].

We thus model the growing niche of non-hybrid rices. Non-hybrid tropical japonica rice requires a high number of GDD to complete its growth. Unlike wheat and barley, germination temperatures for rice are well above freezing (between 18 and 35°C depending on the variety) [39]. Although some seeds have been reported to germinate at temperatures below 10°C, in experimental studies most seedlings either rotted or were not able to establish growth and germination proceeded very slowly [39, 40]. Experimental studies have been carried out to determine the growing degree days of different types of modern rice cultivars [41]. There is a lot of variation among different varieties of rice currently grown in China. For early ripening varieties of tropical *O. japonica*, which ripen in 120 days for a growing degree base of 10°C, an accumulated GDD of 2900 is required. For middle ripening varieties, with a growing season of 120–130 days, an accumulated GDD of 3000–3300 is required. Late maturing species on the other hand with a growing season longer than 130 days require an accumulated GDD of over 3300 [41]. Varieties of *O. japonica* rice adapted to northern China (or temperate *O. japonica*) have slightly lower temperature requirements. For early ripening varieties with a growing season of shorter than 130 days, 2500 GDD are required. For those with a 130–150 day season, 132 between 2500–3000 GDD are required. For late ripening varieties with growing seasons longer than 150 days, over 3000 GDD are required. We model the niche for the minimum requirements of both temperate and tropical *O. japonica* at these different temperature perturbations.

### Data and interpolation

In order to calculate available GDDs across our study area, we use data from the Global Historical Climate Network (GHCN; [42, 43]) from 1961–1990, following Hijmans et al. [44] and Marcott et al. [23]. Daily data from 1,167 weather stations across Asia (all stations within 5°–55° N and 65°–150° W) were screened for joint daily minimum and maximum temperature data, and annual records were removed that had missing data gaps longer than a week. Gaps of a week or less were interpolated from surrounding data using linear interpolation, and only stations with at least ten years of data (again, from 1961–1990) were retained. After data cleaning and standardization, 694 stations remained for analysis. We calculated mean annual climatology by fitting a periodic generalized additive model from minimum and maximum temperature data, independently, using cyclic cubic regression splines for smoothing [45, 46]. Mean daily GDDs were calculated from the daily averages using the equation above (using a *T*_base_ of 10°C), and GDDs were summed over the entire year. Elevations for each weather station were extracted from the Shuttle Radar Topography Mission (SRTM) 1 arc-second global digital elevation model, version 4 [47].

We interpolated annual GDDs (and annual coefficients of variation) by co-Kriging [48]. The variogram was fit to annual GDDs at each weather station using an exponential function of the form *σ* ^2^+*ρ*(1 − exp(−*x*/*θ*)) with *σ* ^2^ the nugget parameter, *ρ* the sill, *θ* the range, and *x* the means of binned great-circle distances between weather stations. After model fitting, interpolation was performed over the 1 arc-minute resolution ETOPO1 digital elevation map [6] for the Shandong study area (33°–40° N and 112°–124° W). All GDD calculations and interpolation were performed in R using the FedData [49] and Fields [48] packages; R scripts detailing all analyses are available as Supplementary Information S1 and S2 Files.

### Temperature perturbation

In order to examine the effects of climatic change during the Holocene we applied a series of temperature perturbations to our data (recalculating GDD and re-interpolating after each perturbation). There is no clear consensus on paleoclimate regimes in Northeastern China during this period of time. Overall, temperatures during the Holocene climatic optimum (c. 7000–3000 cal. BC) were warmer than those of the second half of the 20th century [23]. According to speleothem records, a steady decline in monsoonal intensity that is presumably associated with cooling temperatures takes place throughout the Holocene [50]. Based on pollen biome data, Shi and colleagues [51] estimate that during the Holocene climatic optimum temperatures in North Eastern China may have been up to 3°C higher than modern temperatures. In eastern Hebei province, the climatic optimum is estimated as being up to 4°C higher than modern [52]. Similarly high estimates for temperatures during the climatic optimum have been derived from southern Liaoning province [53]. Here, temperatures derived from pollen biomes are expected to reflect conditions that are 3°C higher than modern at the peak of the climatic optimum.

Global and regional data tell different stories about the timing of climatic change. The GISP ice core record documents an abrupt cooling trend between 3000–2000 cal. BC that follows a period of warmth [54]. A small warming trend begins by roughly 2700 and ends at roughly 2000 cal. BC. This change horizon may correspond with the cooling episode described in world-wide records between ca. 2200–1700 cal. BC that forms the Mid–Late Holocene climate transition [55]. This is followed by a warming period between 1700–1100 cal. BC. Following a longer cooler period, another short warmer burst occurs during the first centuries AD. If anything, the GISP record seems to indicate that temperatures during the mid Holocene were anything but stable and rather fluctuated quite dramatically.

A study of global temperature change tells a somewhat different story [23]. Marcott and colleagues [23] use 73 overlapping climate records that are derived largely from sediment cores from lake bottoms and sea floors, along with data from ice cores. Past temperatures are inferred from the ratio of magnesium and calcium ions (Mg/Ca); or from alkenones (UK 37). This study found that global average temperatures rose until they reached a plateau between 7550 and 3550 cal. BC (the Holocene Climatic Optimum), however they estimate that temperatures were only 1.2°C higher than present during the height of this period. Following this, their data implies that a steady, but long-term cooling trend set in, reaching its lowest temperature extreme between AD 1450 and 1850.

The usefulness of this study for inferring past temperatures in China, must, however, be looked at with scrutiny. A total of 7 ocean cores from Eastern Asia and Island SE Asia are used in the study to infer temperature. Each of these cores tells a very different story in terms of temperature. Alkenone Cores 17940, 18287-3 that are situated in the south China Sea and show a steady decline in temperature from the Holocene climatic optimum that is consistent with the overall pattern shown in Marcott [23]. The two records closest to the Shandong Peninsula KY07-04-01 (Mg/Ca) and A7 (Mg/Ca) however show a very different pattern and show large fluctuations in temperature over the course of the Holocene of an order of approximately 2°C. It is also worth noting that Alkekone cores reflect sea surface temperature (SST). Because of the thermal properties of water, temperatures in the ocean are shown to fluctuate less than those on land. It is likely that temperature fluctuations on land and close to these cores would have exhibited variability on a higher order of magnitude. KY07-04-01, the closest core to Shandong shows a warm period of highly fluctuating temperature between 4000–3700 BC [56]. Following this, a short but marked cooling trend is present at around 3700–3500 BC. From about 3500–2600 another warming trend is present that is interrupted by an abrupt cooling c. 2500. From 2500–1200/1000 BC a relatively stable warming trend seems to be in place [56].

A recent pollen core from Qingdao in Shandong brings more data to the discussion [57]. According to this core, the Holocene climatic optimum in the area would have dated to 5250–3900 cal. BC. Between 3900–2450 cal. BC, a small decline in temperature appears to have taken place, although the flora revealed that temperatures were still on average warmer than those of today. From roughly 2450 cal. BC, arboreal pollen experiences a decline, indicating that a distinct period of climatic cooling took place. A similar climate event has also been recognized in previous pollen data from the Taishizhuang site near Beijing [58]. Here warmer temperatures appear to be in place until about 2400 cal. BC, following which a slight cooling trend appears to be in place 1500 cal. BC, when temperatures begin to cool towards modern levels [58].

Disagreements in pollen records about the timing and amplitude of temperature changes may be due to time lagging responses of perennial plants to climatic changes in local ecosystems.

Due to these uncertainties, we carried out a series of temperature perturbations that ranged from the +4°C predicted by Li and Liang [52]. We found however that this perturbation increases the range of rice across East Asia to one that is entirely too large and thus decided to carry out this experiment at the range of temperatures predicted by Marcott and colleagues [23] and by the sea surface temperature (+2°C). We carry out these perturbations at 0.5°C increments.

## Results and Discussion

The presence of rice at Houli sites was initially confusing to many researchers as the range of rice as modern populations of wild rice do not extend beyond the Yangtze River valley. Today, wild *O. rufipogon* is only distributed in southern China [59]. As a result, doubt was initially cast on the reliability of these remains. Direct AMS dating of these remains, however, prove that these finds date to the 5th millennium cal. BC [9]. It was thus argued that rice may have been traded into the site or imported from areas further to the south [9].

**Fig 3:**
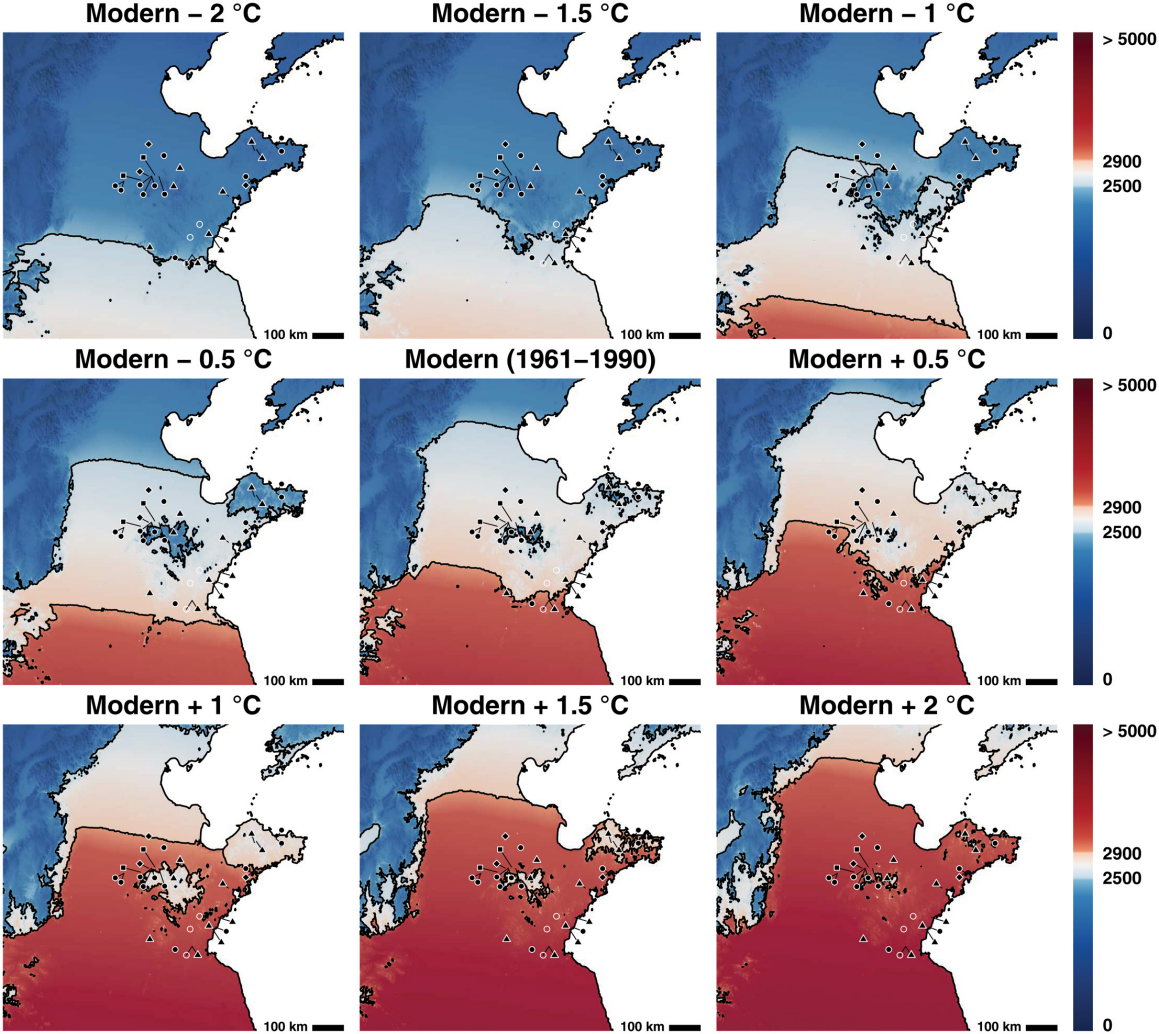
The thermal niche of temperate and tropical *O. japonica* at a series of different temperature perturbations. The area in red represents the area in which tropical *O. japonica* can be grown. The area in white represents the area in which temperate *O. japonica* can be grown. Sites represented by black dots are sites where only millet has been recovered from the assemblage. Sites with a black square represent sites where rice has been unearthed in contexts dating to 6000–5000 cal. BC. Sites with an open circle are sites where rice has been unearthed between 5000–3000 cal. BC, however come from poor or undated contexts. Sites with a black triangle represent finds of rice that date between 2600–2000 cal. BC. Sites with a diamond represents sites where rice has been found that post-dates 1800 cal. BC. Map relief from ETOPO1 [6]; country and province outlines from Natural Earth (naturalearthdata.com).

Our GDD maps show the distribution of temperate and tropical varieties of *O. japonica* under different sets of temperature perturbations. At modern temperatures, tropical varieties of *O. japonica* cannot be grown in Shandong province (Fig 3). However, minor perturbations in temperature could have had a major effect on where rice could be grown. If we follow Marcott [23] in assuming that the climatic optimum was roughly 1°C warmer than the modern (1961–1990) period, then the range of tropical *O. japonica* extends to cover large parts of central Shandong and part of the southern coastline. Houli period sites are within the range for tropical *O. japonica* at this temperature perturbation (Fig 3). It is thus likely that incipient farmers or hunter-gatherers took advantage of wild populations of rice that grew in the area. Although our modeling demonstrates that it would not have been necessary for them to engage in trade to obtain this grain, Chi has argued that cultural connections with the Huai river valley suggest that rice farmers from areas to the south may have moved grain into Shandong [13]. It has been suggested that incipient farmers or hunter-gatherers who exploited rice may have moved into Haidai region from the Huai River valley and Yellow River valleys as extensive evidence of close cultural contact exists between these two regions [60–63].

Following 5000 BC, our review reveals that only a few scant finds of rice are present. These seem to be entirely confined to the southern part of the province and those from dated contexts date to earlier than the end of the optimum (c. 4000 cal. BC) [23]. So why did rice from all but southernmost Shandong between 5000–2600 cal. BC?

The climatic optimum is presumed to have lasted until roughly 3900 cal. BC. Between 3900–2450 cal. BC, a small decline in temperature appears to have taken place, although pollen studies reveal that temperatures were still on average warmer than those of today [57]. It is only from 2450 cal. BC, that temperatures appear to truly cool towards modern levels. At temperature anomalies below +1°C (from modern temperatures), tropical *O. japonica* completely loses range in the area occupied by Houli sites. At modern temperatures, the tropical *O. japonica* niche recedes from Shandong except for in the very most southern portion of the province (Fig 3); indeed, even at temperature anomalies of +0.5°C, the range of rice retreats to the far south of the province (Fig 3).

The initial (and lower amplitude) decline in temperature from Holocene climatic optimum levels thus appears to have had an important impact on wild or early domesticated rices in the area, possibly leading to their retreat away from Central and Northern Shandong [32]. Although some textual evidence from the Song dynasty suggests that wild rice populations may have remained present in the area until AD 1000 [32, 64, 65], our modeling implies that if present it is unlikely that these plants were a tropical variety (Fig 3). Middle Holocene sites that contain evidence of rice in Southern Shandong remain within the niche for tropical *O. japonica* at +0.5°C, and all but one site fall out at modern temperatures (Fig 3). While these mid Holocene finds of rice need to be radiocarbon dated in order to determine that they are not intrusive, these finds are nonetheless located within the range of tropical *O. japonica* during this period of time. These changes likely put considerable pressure on rices that grew in this southern part of the region to adapt to newer and cooler climatic conditions.

Other reasons aside from being locally grown may also explain the presence of rice in Southern Shandong during this period. Between 4300–3000 cal. BC, Shandong becomes increasingly in contact with areas to the south and west. A rise of social complexity, associated with the Dawenkou culture begins to take form in the Taiyi Mountains to the south of Shandong around 4000 cal. BC. This culture gradually spreads to Shandong, northern Jiangsu, northern Anhui and Eastern Henan [66]. Much like in the previous period, areas to the south of Shandong appear to have played an important role in the development of cultural history in this province. It has been suggested that the Dawenkou culture may represent a northward population movement by inhabitants of the Longqiuzhuang culture of Jiangsu province [66]. Development of increasingly complex burial ritual and stratification in the numbers and quality of burial goods is evident during this period of time. This increased contact which goes hand in hand with the development of social complexity makes it possible that longer distance exchange networks could explain the presence of rice at sites in southern Shandong.

During this period of time, other transformations were taking place in populations of rice located further to the south (and possibly even in Shandong). Fuller [32] has argued that there is a relationship between rice morphology and environments. In particular, shorter grains tend to be found at high latitudes or high altitudes, and are typical of temperate *O. japonica* whereas tropical *O. japonica* tends to have longer grains. In the lower Yangtze, differentiation of short-grained rices of temperate morphology and long grained rices of a more tropical/basal morphology appears to have taken place between c. 5000–2250 cal. BC [8, 32]. The same short grained morphology associated with temperate *O. japonica* begins to appear at sites such as Nanjiaokou and middle Yangshao sites located in western Henan from c. 4000–3000 cal. BC [32]. It thus appears that the cooling trend between 3900–2450 cal. BC, may have put adaptive pressure on tropical *O. japonica* rices and led to the development of temperate *O. japonica* rices that were capable of dealing with these cooler temperatures. Whether developed locally or further to the south, these temperate varieties allowed rice to regain its niche in Shandong. Available grain metrics indicate notably short-grained forms of rice in Shandong during the Longshan are consistent with a temperate japonica lineage [8].

Rice re-appears in Shandong assemblages following the beginning of the Longshan period (c. 2600 BC). The re-appearance of rice is linked to the appearance and spread of new cultural material and accompanying demographic transition in the area. From 547 known sites in the Dawenkou period, numbers of sites rise to 1492 during the Longshan period [67]. In Southeastern Shandong, full coverage survey indicates that a process of site nucleation took place with a focus on two large polities: Liangchenzhan (272 ha) and Yaowangcheng (367 ha) [68], where specialized craft production of stone tools and “eggshell” pottery appears to have been a focus of economic activities. Throughout the Longshan period, burial ritual becomes increasingly focused on ancestor worship. Burials containing large amounts of pottery are often accompanied by series of “sacrificial pits” where animal remains are deposited.

Initially sites containing rice appear to cluster around the southeastern part of the Shandong Peninsula, an area that still likely maintained sufficient temperatures for growing even tropical *O. japonica*. At the site of Liangchengzhan in samples derived from an excavation area of 704 m2 on the southern coast, rice forms roughly 47% of the assemblage, while foxtail millet was the other important crop (Table 1) [18]. Interestingly, the rice grains uncovered from this site were, on average, significantly shorter and squatter (4.0 mm by 2.0 mm) than both other archaeological samples from China and modern rice samples [18]. Crawford suggests that this may be because the rice was grown under stress.

**Table 1:** Sites and botanical evidence.

Site Name	Culture Period	Start Year	End Year	AMS Date on Rice	Systematically Collected	Evidence Type	Rice Count	Foxtail Count	Broomcorn Count	Reference
Rizhao Nantunlin	Beixin	-5000	-4500	no date	no	grain	0		6	[78]
Linshu Dongpan	Beixin	-5000	-800	yes	yes	grain/phytolith	2	1	1	[14]
Linshu Dongpan	Longshan					grain/phytolith	359	37	6	[14]
Linshu Dongpan	Xizhou					grain/phytolith	18	10	3	[14]
Rizhao Xujiacun	Late Dawenkou	-3000	-2500	no date	no	grain	1	2	3	[14]
Rizhao Yaowangcheng	Longshan	-2600	-1900	associated	no	grain	0	0	0	[18, 79]
Cangshan Houyuanguanzhuang	Longshan	-2600	-1900	no date	no	grain	0	9	1	[80]
Liangchenzhan	Longshan	-1950	-1950	yes	yes	grain/phytolith	458	499	13	[18]
Juxian/Jiaozhou	Late Dawenkou/Early Longshan	-3000	-2400	no date	no	grain/phytolith	4	2	0	[81]
Zhucheng Xuejiazhuang	Longshan	-2600	-2000	no date	no	grain	4	17	6	[82]
Jining Yuhuangding	Beixin	-5000	-4000				0	0	0	[11]
Zhuanglixi	Longshan	-2600	-1900	associated	no	grain	0	0	0	[18, 83]
Tongling	Longshan	-2600	-1900	associated	yes	grain	8429	12005	15355	[5]
Tongling	Yueshi	-1800	-1600	associated	yes	grain	185	1757	302	[5]
Zhangdian Fangjia	Longshan	-2600	-1900	no date	yes	grain	4	116	111	[84]
Gaoqing Chenzhuang	Early Western Zhou	-1100	-1000	no date	yes	grain/phytolith	1	24246	1420	[85]
Xihe	Houli	-6070	-5900	yes	yes	grain	74	2	0	[10]
Yuezhuang	Houli	-6000	-6000	AMS	yes	grain	26	1	40	[9]
Jinan Pengjia	Yueshi	-1800	-1700	no date	yes	grain	0	67198	2711	[86]
Ma’an	Yueshi	-1800	-1700	no date	yes	grain	0	693	23	[74]
Daxingzhuang	Shang	-1500	-1200	no date	yes	grain/phytolith	44	5602	70	[74]
Jinan Cuimazhuang	Western Zhou	-1100	-1000	no date	yes	grain	0	267	22	[87]
Jinan Tangye	Eastern Zhou	-770	-600	no date	yes	grain	0	1069	18	[88]
Laoling Yinjia	Yueshi	-1800	-1500	no date	yes	grain/phytolith	10	101	40	[89]
Chiping Jiaochangpu	Longshan	-2400	-1900	no date	yes	grain/phytolith	0	292	33	[90]
Chiping Longshan sites	Longshan	-2400	-1900	no date	no	grain/phytolith	0	170	117	[82]
Wenshang Liangzhuang	Ming Dynasty	1350	1400	no date	no	grain	22	49980	25	[91]
Yangjiaquan		-2500	-1850	associated	no	impression	0	0	0	[71]
Yantai Miaohou	Yueshi	-1800	-1600	no date	yes	grain/phytolith	0	66	9	[70]
Yantai Zhaogezhuang	Yueshi	-1800	-1600	no date	yes	grain/phytolith	4	7505	421	[72]
Jiaozhou Zhaojiazhuang	Early to middle Longshan	-2600	-2200	associated	yes	grain/phytolith	425	232	75	[92]
Beiqian	Dawenkou	-3696	-2900	Yes	yes	grain/phytolith	0	36	48	[15]
Beiqian Chunqiu	Chunqiu			Yes	yes	grain/phytolith	11	2043	229	[15]

In far southern Shandong, rice re-appears in Longshan layers at the Linshu Dongpan site, although now in higher proportions and numbers. However, even though our predicted niche estimates that varieties of temperate *O. japonica* could have been grown throughout large parts of Shandong during the Longshan period, the archaeological evidence indicates that the presence of rice as a primary domesticate was confined to the southern coastal area (Fig 3). Large quantities of rice have been unearthed at the Tonglin site in Central Shandong, however in terms of overall proportion, these form roughly 20% of the overall crop assemblage [5]. Rice phytoliths have been unearthed from this site and the Taizigao sites in Northwestern Shandong [17, 69].

**Fig 4:**
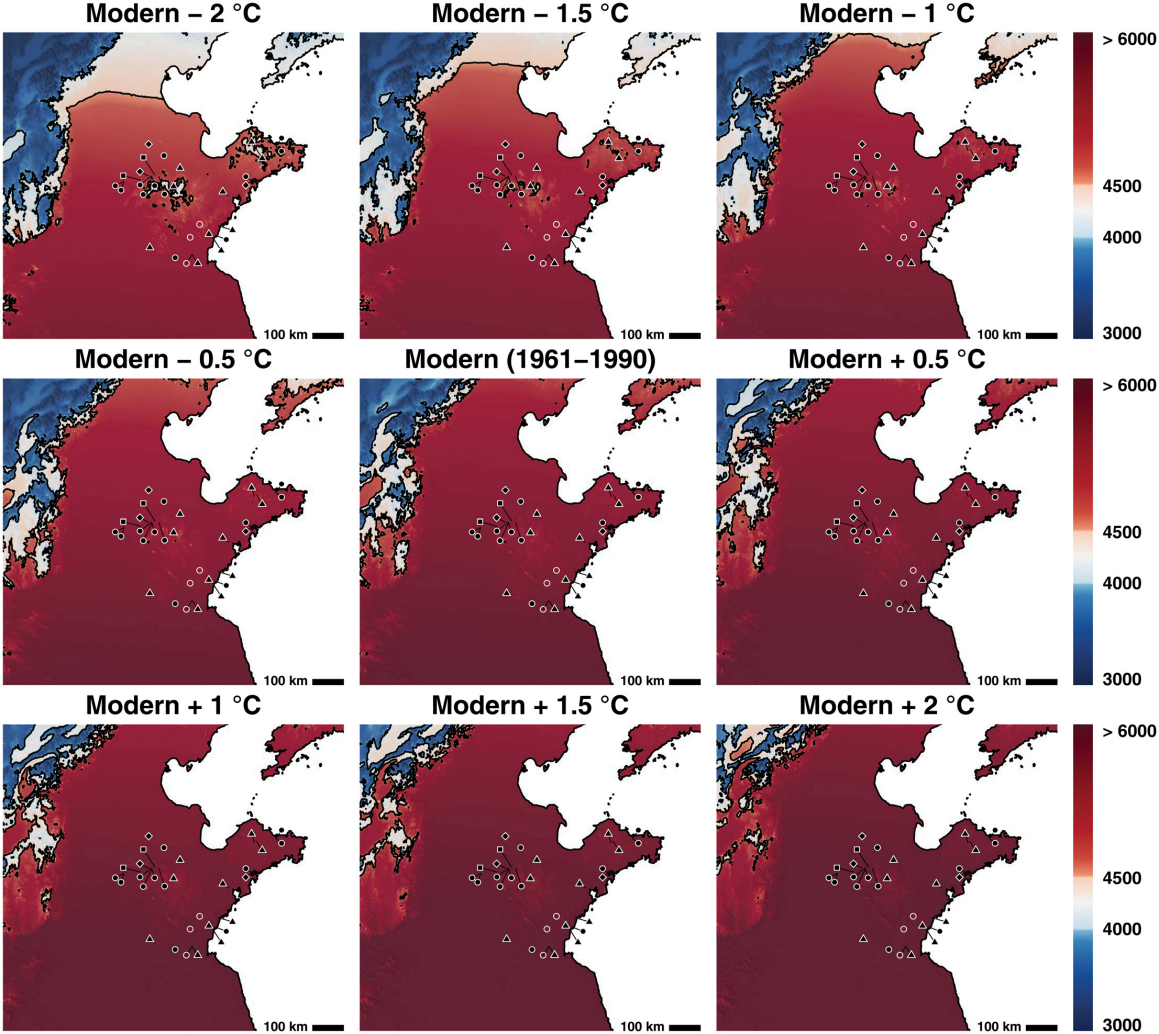
The thermal niche of foxtail millet. At all temperature perturbations it is possible to practice foxtail millet within the area of Shandong. The area in red represents the area in which foxtail can be grown with certainty. The area in white represents the area in cooler adapted varieties of foxtail millet can be grown. Sites represented by black dots are sites where only millet has been recovered from the assemblage. Sites with a black square represent sites where rice has been unearthed in contexts dating to 6000–5000 cal. BC. Sites with an open circle are sites where rice has been unearthed between 5000–3000 cal. BC, however come from poor or undated contexts. Sites with a black triangle represent finds of rice that date between 2600–2000 cal. BC. Sites with a diamond represents sites where rice has been found that post-dates 1800 cal. BC. Map relief from ETOPO1 [6]; country and province outlines from Natural Earth (naturalearthdata.com).

Rice agriculture does not ever appear to have successfully become implanted in the northern Yantai region [70]. Only four grains of rice were recovered from this area despite systematic recovery campaigns. Slightly to the East, rice grain impressions and a single caryopsis have been unearthed at the Yangjiaquan site, however these have not been dated, nor were they derived from systematic excavations [71]. Phytolith evidence at the Yangjiaquan site does however suggest that rice could have been cultivated at the site [71]. At modern temperatures parts of this region were not amenable to growing even temperate *O. japonica*. Millets on the other hand could easily be grown in this area (Fig 4). Longshan sites in the Chiping area of western Shandong appear to have also relied exclusively on millets.

The presence of rice as a primary domesticate appears to last roughly 600 years. Small numbers of finds rice continue, particularly in the southern part of Shandong following 2000 cal. BC. For instance, at Yueshi period sites, such as the Yantai Miaohou and Zhaogezhuang sites, broomcorn and foxtail millet overwhelm the assemblage, indicating that the inhabitants of this area relied on millets [70, 72, 73]. Larger amounts of rice, have, however been unearthed in Yueshi period deposits at the Tonglin site, but in this period decrease to form only 8% of the total crop assemblage [5]. At Daxinzhuang, small numbers of rice have been unearthed, although the diet appears to have been centered primarily around foxtail millet [74, 75]. In particular, following 2000 cal. BC, sites around the central Jinan area seem to transition almost exclusively to millets, suggesting that cultivating rice in this area may no longer have been possible.

If the results of our model provide an accurate guide to past temperatures, this suggests that this well recorded climatic event may have dropped temperatures below that of modern. At -0.5° and -1°C anomalies, both of these areas fall out of or lie on the very border of viability for rice. This contraction of rice niche corresponds with an overall decline in the numbers of sites, a number of which appear to have been abandoned during this period of time. In other areas of China, the period circa 2000 cal. BC appears to have been marked by dramatic climatic cooling and potential drying [22, 76, 77]. Sources disagree, however, as to the magnitude of this event.

**Fig 5:**
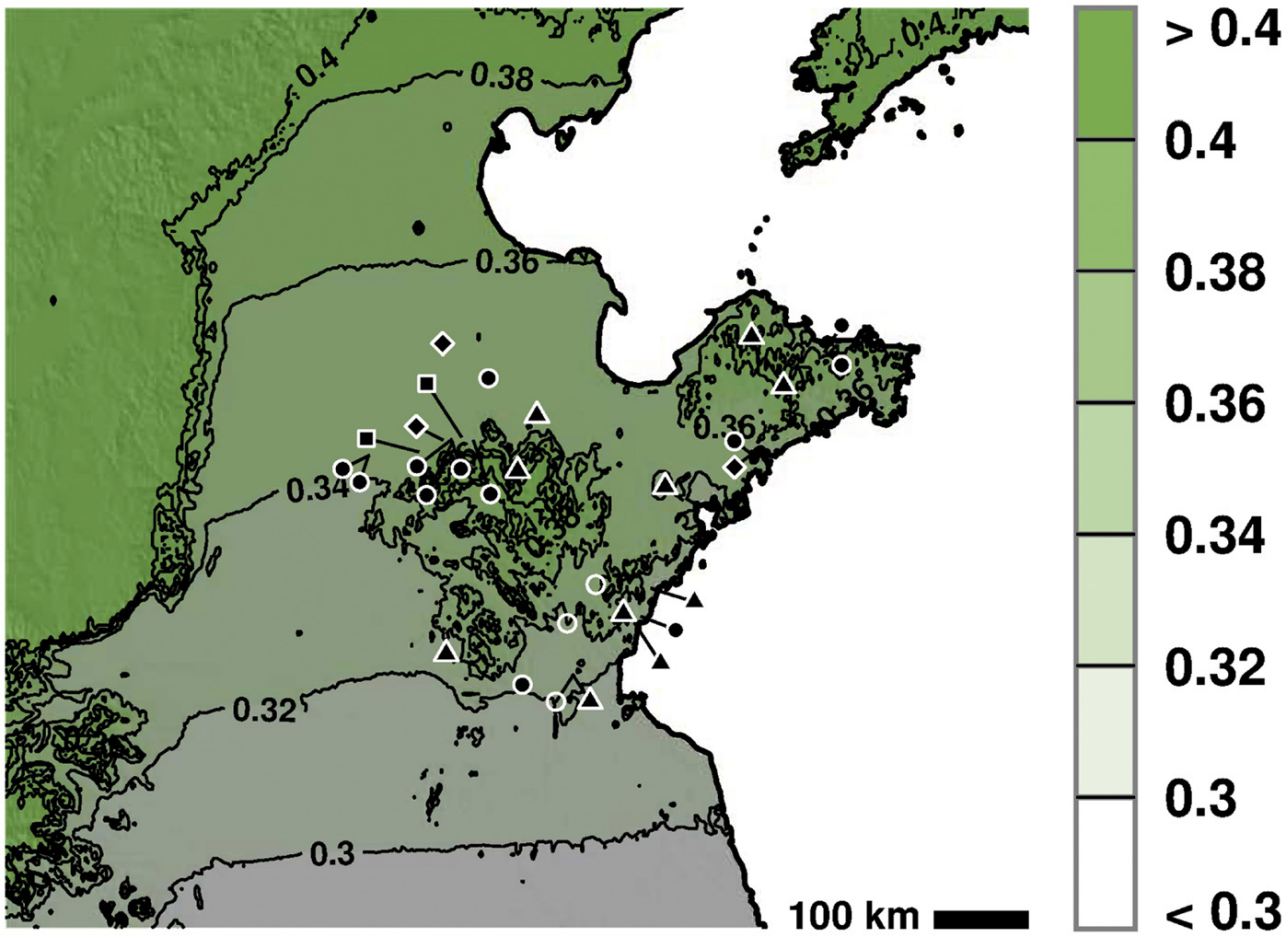
The coefficient of variation in the distribution of growing degree-days across Shandong. Map relief from ETOPO1 [6]; country and province outlines from Natural Earth (naturalearthdata.com).

An examination of the modern (1961–1990) coefficient of variation (CV) of growing degree days across the modern period (in other words an analysis of inter-annual variability of temperatures across the region) demonstrates that the central highlands and the northern part of the peninsula have more inter-annual variability than the lowlands or southern part of the province (Fig 5). When climatic changes took place in these areas, they may have been felt more strongly than at sites in the lowlands. Given the high potential for inter-annual variability in their ability to grow rice, the inhabitants of this region may have opted to shift away from rice cultivation in favor of crops that provided higher protection from risk of lost harvests such as millets.

## Conclusion

Climatic changes during the Holocene played an important role in the spread of rice as a crop across China. Our crop niche model shows that the rice at Houli period sites could have been locally grown or exploited by local hunter-gatherers as tropical varieties of *O. japonica* rice extended its niche into Shandong during the climatic optimum. Relatively minor declines in temperature from the climatic optimum result in tropical *O. japonica* losing its niche in large parts of Shandong. Areas of southern Shandong that lie on the border for of tropical *O. japonica* rices niche may have played an important role in placing adaptive pressure on rice to develop a temperate habit. Once developed, temperate varieties of *O. japonica* facilitated the re-expansion of rice into the province. Cooler areas of the northern peninsula never became fully engaged in rice agriculture. In these cooler areas, the cold tolerant and shorter season crop, foxtail millet appears to have been a primary focus of agricultural production. Ancient farmers in Shandong appear to have adopted rice agriculture in areas and periods of time when it was expedient to do so. Compared to the middle of lower Yangtze, rice does not seem to have played as important a role in the diet. Thanks to their short growing season and ability to tolerate cooler temperatures millets appear to have fulfilled this function.

Climatic conditions have long been cited as important reasons for agricultural change and reorganizations in prehistory, however up until recently the lack of formal modeling applied to these questions has meant that it has been difficult to understand how these changes impacted the distribution of crops on a local scale. There is still, however, much work to be done. At present, we lack a good deep time record of temperature variability across the early Holocene in China. Establishing such a record is key to enabling higher resolution and higher fidelity modeling of the spread of crops across East Asia. By allowing us to clearly outline the set of constraints faced by humans in the past, the use of these models will allow us to arrive at better interpretations of the archaeological record.

## Supporting Information

S1 File **GHCN_PREPPER.R.** This script downloads daily climate records from the Global Historical Climate Network, aggregates those data into an estimate of annual GDD, and interpolates these estimates across a high-resolution raster of a defined area.
(R) Click here for additional data file.

S2 File **SHANDONG.R.** This script creates the figures used in text.
(R) Click here for additional data file.

## References

[1] Bocinsky RK, Kohler TA. A 2,000-year reconstruction of the rain-fed maize agricultural niche in the US Southwest. Nature Communications. 2014;5:5618 10.1038/ncomms6618 25472022

[2] Banks WE, Antunes N, Rigaud S, d’Errico F. Ecological constraints on the first prehistoric farmers in Europe. Journal of Archaeological Science. 2013;40(6):2746–2753. 10.1016/j.jas.2013.02.013

[3] D’Alpoim Guedes J, Butler EE. Modeling constraints on the spread of agriculture to Southwest China with thermal niche models. Quaternary International. 2014;349:29–41. 10.1016/j.quaint.2014.08.003

[4] D’Alpoim Guedes J. Adaptation and Invention during the Spread of Agriculture to Southwest China. Harvard University Cambridge, Massachusetts; 2013.

[5] Song J. The Agricultural Economy During the Longshan Period: An Archaeobotanical Perspective from Shandong and Shanxi. University College London London, England; 2011.

[6] Amante C, Eakins BW. ETOPO1 1 Arc-Minute Global Relief Model: Procedures, Data Sources and Analysis NOAA Technical Memorandum NESDIS NGDC-24. National Geophysical Data Center, NOAA; 2009 Available from: http://www.ngdc.noaa.gov/mgg/global/global.html.

[7] Jin G, Luan F, Zhang C, Wang Y. Report on the Investigation of Ancient Agriculture in Southern Liaodong Peninsula: Evidence of Phytolith. Dongfang Kaogu. 2009;6:305–316.

[8] Fuller DQ, Sato YI, Castillo C, Qin L, Weisskopf AR, Kingwell-Banham EJ, et al Consilience of genetics and archaeobotany in the entangled history of rice. Archaeological and Anthropological Sciences. 2010;2(2):115–131. 10.1007/s12520-010-0035-y

[9] Crawford G, Chen X, Wang J. Discovery of fossilized crops from the Houli site of Yuezhuang in the Jinan Region of Shandong. Dongfang Kaogu. 2006;3:247–251.

[10] Jin G, Wu W, Zhang K, Wang Z, Wu X. 8000-year old rice remains from the north edge of the Shandong Highlands, East China. Journal of Archaeological Science. 2014;51:34–42. 10.1016/j.jas.2013.01.007

[11] Jin G, Zhao M, Wang Z, Tang T. A report on the foxtail millet systems of the Longshan: Report at the Yuhuanding site in Jining, Shandong. Haidai Kaogu. 2010;3:100–113.

[12] Hu Y, Wang S, Lu F, Wang C, Richards MP Stable isotope analysis of humans from Xiaojingshan site: Implications for understanding the origin of millet agriculture in China. Journal of Archaeological Science. 2008;35(11):2960–2965. 10.1016/j.jas.2008.06.002

[13] Zhang C, Hung HC. Jiahu 1: Earliest farmers beyond the Yangtze River. Antiquity. 2013;87(335):46–63. 10.1017/S0003598X00048614

[14] Wang H, Liu C, Jin G. Report on the carbonized seeds from the Dongpan site in Linshu County, Shandong. Dongfang Kaogu. 2012;8:357–372.

[15] Jin G, Wang Y. Report on the archaeobotanical remains unearthed from the Beiqian site. Kaogu. 2011;11:19–22.

[16] Wang H, Jin G. Report on the archaeobotanical remains from the site of Beiqian in Jimo, Shandong. Dongfang Kaogu. 2013;10:255–279.

[17] Jin G, Zhao M, Liu Y, He Z. An archaeobotanical survey of Ying County and Jiaozhou in Shandong. Dongfang Kaogu. 2009;6:344–349.

[18] Crawford G, Underhill A, Zhao Z, Lee G, Feinman G, Nicholas L, et al Late Neolithic plant remains from northern China: Preliminary results from Liangchengzhen, Shandong. Current Anthropology. 2005;46(2):309–317. 10.1086/428788

[19] Jin G, Yan S, Udatsu T, Lan Y, Wang C, Tong P. Neolithic rice paddy from the Zhaojiazhuang site, Shandong, China. Chinese Science Bulletin. 2007;52(24):3376–3384. 10.1007/s11434-007-0449-9

[20] Fuller DQ, Qin L. Water management and labour in the origins and dispersal of Asian rice. World Archaeology. 2009;41(1):88–111. 10.1080/00438240802668321

[21] An CB, Tang L, Barton L, Chen FH. Climate change and cultural response around 4000 cal yr BP in the western part of Chinese Loess Plateau. Quaternary Research. 2005;63(3):347–352. 10.1016/j.yqres.2005.02.004

[22] Dong G, Jia X, An CB, Chen FH, Zhao Y, Tao S, et al Mid-Holocene climate change and its effect on prehistoric cultural evolution in eastern Qinghai Province, China. Quaternary Research. 2012;77(1):23–30. 10.1016/j.yqres.2011.10.004

[23] Marcott SA, Shakun JD, Clark PU, Mix AC. A reconstruction of regional and global temperature for the past 11,300 years. Science. 2013;339(6124):1198–1201. 10.1126/science.1228026 23471405

[24] Jones HG. Plants and Microclimate: A Quantitative Approach to Environmental Plant Physiology. Cambridge University Press; 2013.

[25] McMaster GS, Edmunds D, Wilhelm W, Nielsen D, Prasad P, Ascough J II. Phenology MMS: A program to simulate crop phenological responses to water stress. Computers and Electronics in Agriculture. 2011;77:118–125. 10.1016/j.compag.2011.04.003

[26] McMaster GS, Wilhelm WW. Growing degree-days: One equation, two interpretations. Agricultural and Forest Meteorology. 1997;87(4):291–300. 10.1016/S0168-1923(97)00027-0

[27] Garris AJ, Tai TH, Coburn J, Kresovich S, McCOUCH S. Genetic structure and diversity in *Oryza sativa L* . Genetics. 2005;169(3):1631–1638. 15654106 10.1534/genetics.104.035642 PMC1449546

[28] Londo JP, Chiang YC, Hung KH, Chiang TY, Schaal BA. Phylogeography of Asian wild rice, *Oryza ru pogon*, reveals multiple independent domestications of cultivated rice, *Oryza sativa* . Proceedings of the National Academy of Sciences. 2006;103(25):9578–9583. 10.1073/pnas.0603152103 PMC1480449 16766658

[29] Sweeney M, McCouch S. The complex history of the domestication of rice. Annals of Botany. 2007;100(5):951–957. 10.1093/aob/mcm128 17617555 PMC2759204

[30] Zhao K, Tung CW, Eizenga GC, Wright MH, Ali ML, Price AH, et al Genome-wide association mapping reveals a rich genetic architecture of complex traits in *Oryza sativa* . Nature Communications. 2011;2:467 10.1038/ncomms1467 21915109 PMC3195253

[31] Zhang LB, Zhu Q, Wu ZQ, Ross-Ibarra J, Gaut BS, Ge S, et al Selection on grain shattering genes and rates of rice domestication. New Phytologist. 2009;184(3):708–720. 10.1111/j.1469-8137.2009.02984.x 19674325

[32] Fuller DQ. Pathways to Asian civilizations: Tracing the origins and spread of rice and rice cultures. Rice. 2012;4:78–92. 10.1007/s12284-011-9078-7

[33] Bray F. The rice economies: Technology and development in Asian societies. University of California Press; 1994.

[34] He Z, Bonjean AP. Cereals in China. Mexico: International Maize and Wheat Improvement Center; 2010.

[35] Tang S, Ding L, Bonjean AP. Rice production and genetic improvement in China In: He Z, Bonjean AP, editors. Cereals in China. Mexico: International Maize and Wheat Improvement Center; 2010 p. 15.

[36] Zeng X. Huang Lu Rice in Chinese History. Nongye Kaogu. 1998;1:292–307.

[37] Ho PT. Early-ripening rice in Chinese history. The Economic History Review. 1956;9(2):200–218. 10.1111/j.1468-0289.1956.tb00657.x

[38] Jiang P. Shou Shi Tongkao Quanshu. Daoguang; 1826.

[39] International Rice Research Institute. Weather and Rice. Proceedings of the international workshop on The Impact of Weather Parameters on Growth and Yield of Rice; 1986.

[40] Nishiyama I. Effects of temperature on the vegetative growth of rice plants. Climate and Rice. 1976;p. 159–185.

[41] Rice Growth and Temperature. Beijing: Chinese Institute of Agronomy; 2011.

[42] Menne MJ, Durre I, Korzeniewski B, McNeal S, Thomas K, Yin X, et al. Global Historical Climatology Network-Daily (GHCN-Daily), Version 3; 2012 Available from: 10.7289/V5D21VHZ.

[43] Menne MJ, Durre I, Vose RS, Gleason BE, Houston TG. An overview of the Global Historical Climatology Network-Daily database. Journal of Atmospheric and Oceanic Technology. 2012;29(7):897–910. 10.1175/JTECH-D-11-00103.1

[44] Hijmans RJ, Cameron SE, Parra JL, Jones PG, Jarvis A. Very high resolution interpolated climate surfaces for global land areas. International Journal of Climatology. 2005;25(15):1965–1978. 10.1002/joc.1276

[45] Wood SN, Augustin NH. GAMs with integrated model selection using penalized regression splines and applications to environmental modelling. Ecological Modelling. 2002;157(2):157–177. 10.1016/S0304-3800(02)00193-X

[46] Wood SN. Fast stable restricted maximum likelihood and marginal likelihood estimation of semiparametric generalized linear models. Journal of the Royal Statistical Society: Series B (Statistical Methodology). 2011;73(1):3–36. 10.1111/j.1467-9868.2010.00749.x

[47] Jarvis A, Reuter HI, Nelson A, Guevara E. Hole-filled seamless SRTM data V4, International Centre for Tropical Agriculture (CIAT); 2008 Available from: http://srtm.csi.cgiar.org.

[48] Nychka D, Furrer R, Sain S. fields: Tools for spatial data; 2014. R package version 7.1. Available from: http://CRAN.R-project.org/package = fields.

[49] Bocinsky RK. FedData: Functions to Automate Downloading Geospatial Data Available from Several Federated Data Sources; 2015. R package version 1.0. Available from: http://CRAN.R-project.org/package = FedData.

[50] Dykoski CA, Edwards RL, Cheng H, Yuan D, Cai Y, Zhang M, et al A high-resolution, absolute-dated Holocene and deglacial Asian monsoon record from Dongge Cave, China. Earth and Planetary Science Letters. 2005;233(1):71–86. 10.1016/j.epsl.2005.01.036

[51] Yafeng S, Zhaozheng K, Sumin W, Lingyu T, Fubao W, Tandong Y, et al Mid-Holocene climates and environments in China. Global and Planetary Change. 1993;7(1):219–233. 10.1016/0921-8181(93)90052-P

[52] Li W, Liang Y. Vegetation and environment in eastern Hebei Province during the Holocene Megathermal. Acta Botanica Sinica. 1985;27(6):640–651.

[53] Chen C, Lu Y, Shen C. Evolution of the natural environment in southern Liaoning province since 10 ka. Science in China (Series D). 1977;p. 603–614.

[54] Alley RB, Gow A, Johnsen S, Meese D, Kipfstuhl J, Thorsteinsson T. Comparison of deep ice cores. Nature. 1995;373:393–394. 10.1038/373393b0 7830789

[55] Walker M, Berkelhammer M, Björck S, Cwynar L, Fisher D, Long A, et al Formal subdivision of the Holocene Series/Epoch: A Discussion Paper by a Working Group of INTIMATE (Integration of ice-core, marine and terrestrial records) and the Subcommission on Quaternary Stratigraphy (International Commission on Stratigraphy). Journal of Quaternary Science. 2012;27(7):649–659. 10.1002/jqs.2565

[56] Anand P, Elderfield H, Conte MH. Calibration of Mg/Ca thermometry in planktonic foraminifera from a sediment trap time series. Paleoceanography. 2003;18(2). 10.1029/2002PA000846

[57] Chen W, Wang WM. Middle-Late Holocene vegetation history and environment changes revealed by pollen analysis of a core at Qingdao of Shandong Province, East China. Quaternary International. 2012;254:68–72. 10.1016/j.quaint.2011.04.005

[58] Tarasov P, Jin G, Wagner M. Mid-Holocene environmental and human dynamics in northeastern China reconstructed from pollen and archaeological data. Palaeogeography, Palaeoclimatology, Palaeoecology. 2006;241(2):284–300. 10.1016/j.palaeo.2006.03.038

[59] Pang H, Chen C. Wild Rice in China. Guangxi Provincial Science and Technology Press; 2002.

[60] Zhang C. On the remains of the first stage of Jiahu Culture. Wenwu. 2011;3:46–53.

[61] Han J. The northward movement of Shuangdun and the formation of Beixin Culture—Evidence from the Beixin Culture remains from Zhangshan site, Jining. Jianghan Kaogu. 2012;2:46–50.

[62] Luan F. Discussing the Houli culture. Haidai Kaogu Yanjiu. 1997;p. 1–26.

[63] Institute of Archaeology of Nanjing Museum, Museum of Sihong County. The excavation of the Shunshanji site of Neolithic Age in Sihong County, Jiangsu. Kaoguxuebao. 2014;4:519–562.

[64] Ho P. The indigenous origins of Chinese agriculture In: Reed C, editor. The Origins of Agriculture. The Hague: Mouton; 1977 p. 413–484.

[65] You X. The wild rice in Chinese ancient records. Gujin Nongye Ancient and Modern Agriculture. 1987;p. 1–6.

[66] Han J. North movement of the Longqiuzhuang culture and the formation of the Dawekou culture. Jianghan Kaogu. 2011;1:59–64.

[67] Liu L, Chen X. The Archaeology of China: from the Paleolithic to the Early Bronze. England: Cambridge University Press; 2012.

[68] Underhill AP, Feinman GM, Nicholas LM, Fang H, Luan F, Yu H, et al Changes in regional settlement patterns and the development of complex societies in southeastern Shandong, China. Journal of Anthropological Archaeology. 2008;27(1):1–29. 10.1016/j.jaa.2006.11.002

[69] Jin G, Lu H, Wei C. A discussion on the phytolith remains from the Longshan period Tianwang site. Kaogu. 1999;2:82–87.

[70] Jin G, Wang Z, Wang F. A report on the plant remains from the Miaohou site in Yantai. Dongfang Kaogu. 2009;6:321–330.

[71] Luan F, Jin G, Wang F. Survey and preliminary study of rice farming remains on the Yangjiaquan site in Qixia County, Shandong. Kaogu. 2007;12:78–84.

[72] Jin G, Zhang M, Wang Z, Wang F, Jiang G. Report on the archaeobotanical remains from the Zhaogezhuang site in Yantai. Dongfang Kaogu. 2009;6:331–343.

[73] Chen X. Preliminary research on agriculture of Yueshi Culture. Dongfang Kaogu. 2012;9:607–625.

[74] Chen X, Fang H. A case study of agriculture in the Shang Dynasty: Macro plant remains from the Daxinzhuang site, Jinan, China. Dongfang Kaogu. 2008;4:47–68.

[75] Jin G, Fang H. Report on the phytoliths from the Daxinzhuang Site. Dongfang Kaogu. 2008;4:30–46.

[76] An CB, Feng Z, Tang L. Environmental change and cultural response between 8000 and 4000 cal. yr BP in the western Loess Plateau, northwest China. Journal of Quaternary Science. 2004;19(6):529–535. 10.1002/jqs.849

[77] An CB, Ji D, Chen F, Dong G, Wang H, Dong W, et al Evolution of prehistoric agriculture in central Gansu Province, China: A case study in Qin’an and Li County. Chinese Science Bulletin. 2010;55(18):1925–1930. 10.1007/s11434-010-3208-2

[78] Chen X. Results on the otation carried out at two sites in the Rizhao area of Shandong. Nanfang Wenwu. 2007;1:92–94.

[79] Unknown. Important discoveries from the second excavations at the Yaowangcheng site. Zhongguo Wenwu Bao. 1994 January;.

[80] Wang H, He D, Jin G. Report on the plant remains from the Houyangguangzhuang site in Cangshan. Haidai Kaogu. 2013;6:133–138.

[81] Jin G, Zhao M, Wang Z, Liu Y, He Z, et al Report on the archaeobotanical survey of Yingxian and Jiaozhou Shandong. Dongfang Kaogu. 2009;6:344–349.

[82] Jin G, Zhao M, Sun Z, Sun J. A report on the archaeobotanical survey on the Chiping area Longshan Sites. Dongfang Kaogu. 2009;6:317–320.

[83] Kong Z, Liu C, He D. The significance from the perspective of environmental archaeology of plants preserved at the site of Zhuanglixi in Tengzhou City, Shandong. Kaogu. 1999;7:59–62.

[84] Jin G, Wang Z, Zhang K, Wang Z. Report on the archaeobotanical remains from the Longshan Period site of Fangjia in Zibo City. Haidai Kaogu. 2011;4:67–72.

[85] Jin G. Report on the carbonized seeds and fruits from the site of Gaoqing Chenzhuang in Shandong. Nanfang Wenwu. 2012;1:147–155.

[86] Wu W, Lei D, Jin G. Jinan Pengjiazhuang Yizhi Fuxuan Jieguo Chubu Fenxi. Dongfang Kaogu. 2010;7:359–369.

[87] Wu W, Han J, Jin G. A report on the archaeobotanical remains from the Majiazhuang site in Jinan. Dongfang Kaogu. 2010;7:351–357.

[88] Zhao M, Chen X, Gao X, He L. Shandong Sheng Jinan Shi Tangye Yizhi Fuxuan Jieguo Fenxi. Nanfang Wenwu. 2008;2:120–125.

[89] Zheng X, Zhu C, Wang H, Xu Q. Report on the archaeobotanical remains from the Yueshi site of Laoling Yingjia in Shandong. Haidai Kaogu. 2013;6:139–150.

[90] Zhao Z. A comparison of the archaeobotanical remains between the Liangchenzhan and Jiaochangpu. Dongfang Kaogu. 2004;1:210–224.

[91] Wu W, Yan X, Gao M, Jin G. Report on the archaeobotanical remains from the Liangzhuang site in Wenshang, Jining. Dongfang Kaogu. 2010;7:370–378.

[92] Jin G, Ge G, Wang H, Yan S, Liu Cea. Report on the carbonized plant remains from the Longshan period Zhaojianzhuang site in Jiaozhou, Shandong. Keji Kaogu. 2011;3:37–53.

